# Advanced quantitative analysis of the sub-retinal pigment epithelial space in recurrent neovascular age-related macular degeneration

**DOI:** 10.1371/journal.pone.0186955

**Published:** 2017-11-02

**Authors:** Mariko Sasaki, Yu Kato, Kaoru Fujinami, Toshiaki Hirakata, Kazushige Tsunoda, Ken Watanabe, Kunihiko Akiyama, Toru Noda

**Affiliations:** 1 Department of Ophthalmology, National Institute of Sensory Organs, National Tokyo Medical Center, Tokyo, Japan; 2 Department of Ophthalmology, Tachikawa Hospital, Tokyo, Japan; 3 Department of Ophthalmology, Keio University School of Medicine, Tokyo, Japan; 4 Department of Ophthalmology, Metropolitan Komagome Hospital, Tokyo, Japan; 5 Genetics, UCL Institute of Ophthalmology, London, United Kingdom; University of Manchester, UNITED KINGDOM

## Abstract

To quantitatively evaluate changes in the sub-retinal pigment epithelial (RPE) space and determine the association with recurrent neovascular age-related macular degeneration (AMD). Twenty-two eyes treated with intravitreal aflibercept for treatment-naïve neovascular AMD were studied retrospectively. The sub-RPE area, volume, and central retinal thickness (CRT) were evaluated 1 and 2 months after the loading phase using spectral-domain optical coherence tomography. Recurrence was defined as newly detected neovascular activity during the 6 months after the loading phase. In eyes with recurrent AMD, the sub-RPE area increased significantly (*P* = 0.036) from 1 to 2 months after the loading phase and the sub-RPE volume increased marginally (*P* = 0.06). Subgroup analysis showed significant (*P* = 0.008 and *P* = 0.016, respectively) increases in the sub-RPE area and volume in typical AMD. In eyes with no recurrence, no significant changes occurred in the two parameters. No significant CRT changes occurred in eyes with or without a recurrence. A quantitative analysis demonstrated an increased likelihood of the sub-RPE space shortly after the loading phase in eyes with recurrent AMD; no changes occurred in eyes without a recurrence. These early changes in the sub-RPE space could indicate disease activity and are valuable for predicting recurrences of neovascular AMD.

## Introduction

Age-related macular degeneration (AMD) is a leading cause of visual loss worldwide.[1] The introduction of anti-vascular endothelial growth factor (VEGF) treatment dramatically reduced the prevalence of legal blindness and visual impairment due to AMD.[2] However, anti-VEGF therapy does not cure AMD, and the primary goals of maintenance therapy are achieving control of disease activity and avoiding recurrences with minimal substantial sensory retinal impairment. Therefore, relevant parameters that indicate disease activity and predict recurrences are mandatory to establish better treatment practices for AMD.

Choroidal neovascularization (CNV) originates from the choroid, penetrates Bruch’s membrane, and develops into the subretinal or sub-retinal pigment epithelial (sub-RPE) space, with accompanying exudative changes involving intraretinal, subretinal, and sub-RPE fluid and hemorrhages.[1, 3] RPE elevation and enlargement of the sub-RPE space result from fluid, hemorrhages, or the neovascular component itself. One of these, retinal pigment epithelial detachment (PED), is considered associated with disease activity [4–6]. We hypothesized that changes in the sub-RPE space are relevant parameters of disease activity that might indicate recurrences before substantial changes occur in the sensory retina.

Advanced RPE analysis quantitatively measures the sub-RPE space using optional software in the Cirrus spectral-domain optical coherence tomography instrument (SD-OCT, Carl Zeiss Mediate, Inc., Dublin, CA).[7] The software automatically segments the RPE and virtual RPE floor, reconstructs three-dimensional images, and provides the area and volume of the sub-RPE space.

We sought to quantitatively measure the changes in the sub-RPE space shortly after the loading phase of anti-VEGF treatment in patients with neovascular AMD to determine an association with recurrent disease during a 6-months period and compare the sub-RPE changes with the appearance of the sensory retina. We also investigated the relationship between the sub-RPE changes and the presence of recurrences based on the subtype of the neovascular AMD.

## Methods

### Study population

The Institutional Review Board of the National Hospital Organization, Tokyo Medical Center, approved this retrospective consecutive case series (R14-122), which adhered to the tenets of the Declaration of Helsinki. All patients with treatment-naïve neovascular AMD seen between January 2013 and August 2014 at the Department of Ophthalmology, National Hospital Organization, Tokyo Medical Center, were reviewed retrospectively.

The main eligibility criteria included three consecutive monthly intravitreal injections of 2.0 mg of aflibercept (Eylea, Regeneron Pharmaceuticals, Tarrytown, NY) for treatment-naive AMD and a minimum of 6 months of follow-up after the loading phase using a pro re nata (PRN) regimen with monthly evaluations. If both eyes of a patient were eligible for study analysis, the eye treated first was included. Patients were excluded if they had any one of the following: 1) if a dry macula, defined as the absence of intraretinal or subretinal fluid, was not achieved; 2) inadequate quality of the OCT images, i.e., unclear images or inappropriate segmentation due to an excessively large PED or fibrosis; 3) receipt of treatment approaches other than anti-VEGF therapies, such as photocoagulation or photodynamic therapy, within 6 months after the loading phase.

### Assessment of neovascular AMD

Fluorescein angiography (FA) and indocyanine green angiography (ICGA) (HRA-2, Heidelberg Engineering Inc., Heidelberg, Germany) were performed to determine the AMD subtypes before the initial treatment. All cases were classified into one of three AMD subtypes according to the funduscopic and angiographic (FA and ICGA) findings: typical AMD,[8] polypoidal choroidal vasculopathy (PCV),[9] and retinal angiomatous proliferation (RAP).[10] Typical AMD was defined as the presence of serous or hemorrhagic exudative changes or both and consistent CNV detected on FA and ICGA images.[8] PCV was defined as the presence of elevated orange-red lesions observed by fundus examination and/or characteristic polypoidal lesions on ICGA.[9] RAP was diagnosed based on the classification of Yannuzzi and associates.[10] The subtype classifications were made based on the agreement of all co-authors before the treatment began.

Intraretinal fluid (IRF) was defined as having round, minimally reflective spaces within the retina. Subretinal fluid (SRF) was identified as a nonreflective space between the posterior boundary of the retina and the RPE. PEDs were defined based on the OCT measurements: radial extension of an RPE elevation at the base >400 μm or the vertical elevation from the RPE to the surface of the choriocapillaris >200 μm[11]. Sub-types of PEDs were differentiated to serous PEDs (sPED), fibrovascular PEDs (fPED) or hemorrhagic PEDs (hPED) according to the OCT and FA features.

### Management of neovascular AMD

Patients received three or more consecutive monthly intravitreal injections of 2.0 mg of aflibercept until no signs of CNV activity were detected and then were retreated using a flexible PRN regimen with monthly evaluations for interim injections.[4] CNV activity was defined as the presence of intraretinal/subretinal fluid detected by SD-OCT or macular hemorrhages.[11] OCT imaging was performed at each visit, and a macular cube protocol was applied for the purpose of analyses.

### Measurements of changes in the sensory retina and sub-RPE space

Advanced RPE analysis quantitatively measures the sub-RPE space using optional software in the Cirrus spectral-domain optical coherence tomography instrument (SD-OCT, Carl Zeiss Mediate, Inc., Dublin, CA).[7] The software automatically segments the RPE and virtual RPE floor using a segmentation algorithm, reconstructs three-dimensional images, and provides the area and volume of the sub-RPE space. Eyes were scanned routinely at each visit by Cirrus SD-OCT using the 512 × 128 macular cube protocols covering a 6 × 6-mm area centered on the fovea. The sub-RPE area and volume (within a 5-mm circle) and central retinal thickness (CRT, within a 1-mm circle) were evaluated 1 and 2 months after the loading phase.

Recurrences were defined as newly detected CNV activity within 6 months after the loading phase (i.e. early recurrence). Non-recurrent cases were defined as those without a recurrence within 6 months after the loading phase (i.e. late or no recurrence generally).

All scans were reviewed, and one (YK) co-author who was masked to the patients’ clinical details excluded the poor-quality scans including inadequate segmented lines as described previously.

### Statistical analysis

The baseline characteristics are presented for the overall sample, and subgroups were stratified based on recurrences of AMD. The data are expressed as the mean or mean ± standard deviation. Differences in the basic characteristics between cases with and without a recurrence were assessed using the Wilcoxon rank-sum test for continuous variables and the chi-square test or Fisher’s exact test for categorical variables. To compare the sub-RPE area and volume and the CRT between 1 and 2 months after the loading phase, the Wilcoxon signed-rank test was applied. A *P*-value less than 0.05 was considered significant. The positive and negative predictive values were calculated to investigate the association of changes in the sub-RPE area between 1 and 2 months after the loading phase and recurrences in 6 months.[12] All analyses were performed using SAS version 9.4 for Windows (SAS Institute Inc., Cary, NC).

## Results

**Fig 1:**
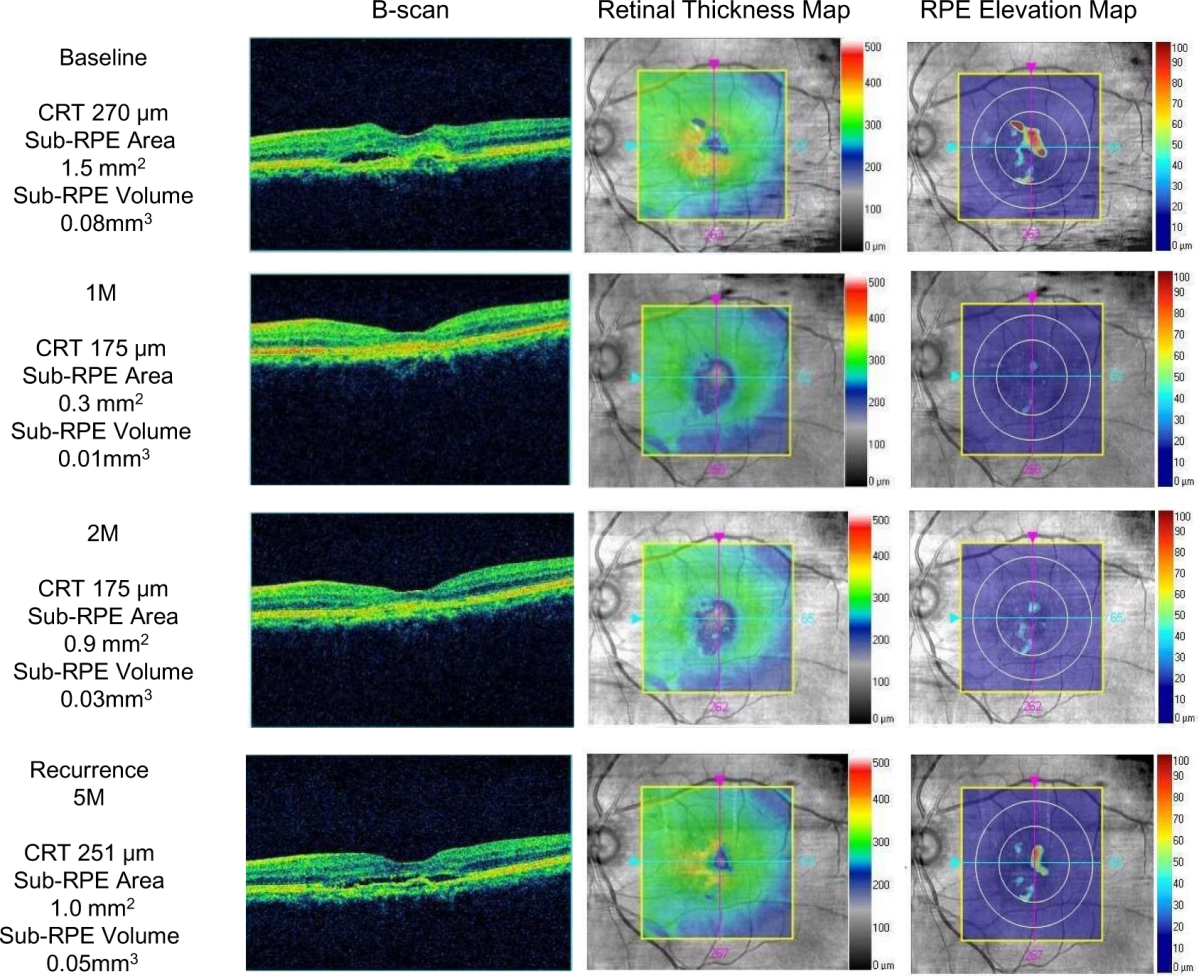
Spectral-domain optical coherence tomography imaging obtained from an 82-year-old man with type 1 choroidal neovascularization. (Top row) At baseline, the B-scan shows a sub-RPE space with subretinal fluid and subretinal hyperreflective material. The RT map shows the presence of fluid (orange); the central retinal thickness (CRT) is 270 microns. The RPE elevation map shows the sub-RPE space (red); the sub-RPE area and volume are 1.5 mm^2^ and 0.08 mm^3^, respectively. (Second row) One month after the loading phase, the subretinal fluid has disappeared from the B-scan and RT map, and the CRT has decreased to 175 microns. The RPE elevation map shows the decreased sub-RPE space; the area and volume are 0.3 mm^2^ and 0.01 mm^3^, respectively. (Third row) Two months after the loading phase, the B-scan and RT map are stable compared to the previous month; the CRT is unchanged at 175 microns. In contrast, the RPE elevation map shows a considerably larger pigment epithelial detachment (PED) compared with the previous month; the area and volume are 0.9 mm^2^ and 0.03 mm^3^, respectively. (Bottom row) At the time of recurrence at 5 months after the loading phase, the B-scan shows that a sub-RPE space developed with subretinal fluid. The RT map shows the presence of fluid again (yellow), and the CRT has increased to 251 microns. The RPE elevation map shows an increase in the area of the sub-RPE; the area and volume are 1.0 mm^2^ and 0.05 mm^3^, respectively.

Thirty-four eyes met the eligibility criteria, 12 of which were excluded because of an extended interval between visits in seven eyes, no achievement for a dry macula in one eye, inadequate OCT image quality in two eyes, and other treatment approaches in two eyes. Twenty-two eyes that met the inclusion and exclusion criteria were analyzed (14 men, 63.6%; 8 women, 36.4%; average age, 74.2±8.9 years). All demographic information and retinal and sub-RPE measurements during the study period were available for all cases. Regarding the AMD subtypes, 12 eyes (54.5%) had typical AMD, seven eyes (31.8%) PCV, and three eyes (13.6%) RAP. Thirteen (59.1%) patients had a recurrence, and nine (40.9%) patients did not. The number of the injection to achieve dry macula was 3.1±0.6, and the average time to a recurrence was 3.3 months (range, 2–5 months). Fig 1 shows a typical case with a recurrence. There were no significant demographic differences between eyes with and without a recurrence in the retinal or sub-RPE qualitative assessment or measurements at baseline. Persistent PEDs were found 3 cases in the recurrence group and 2 cases in the non-recurrence group 1 and 2 months after the loading phase.

**Fig 2:**
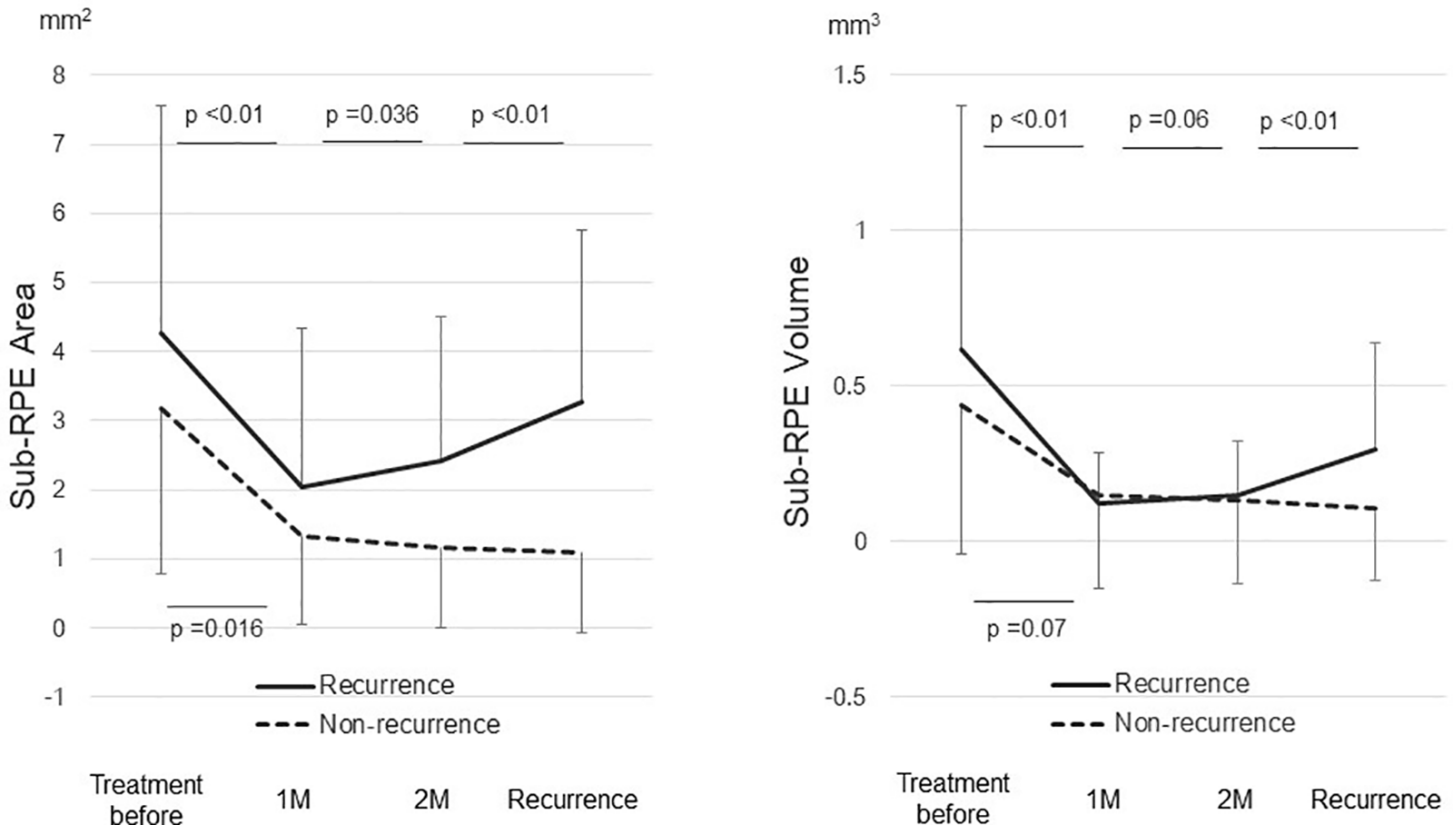
Changes of the sub-RPE area and volume in all cases after aflibercept therapy. 1 M = 1 month after the loading phase; 2 M = 2 months after the loading phase.

**Fig 3:**
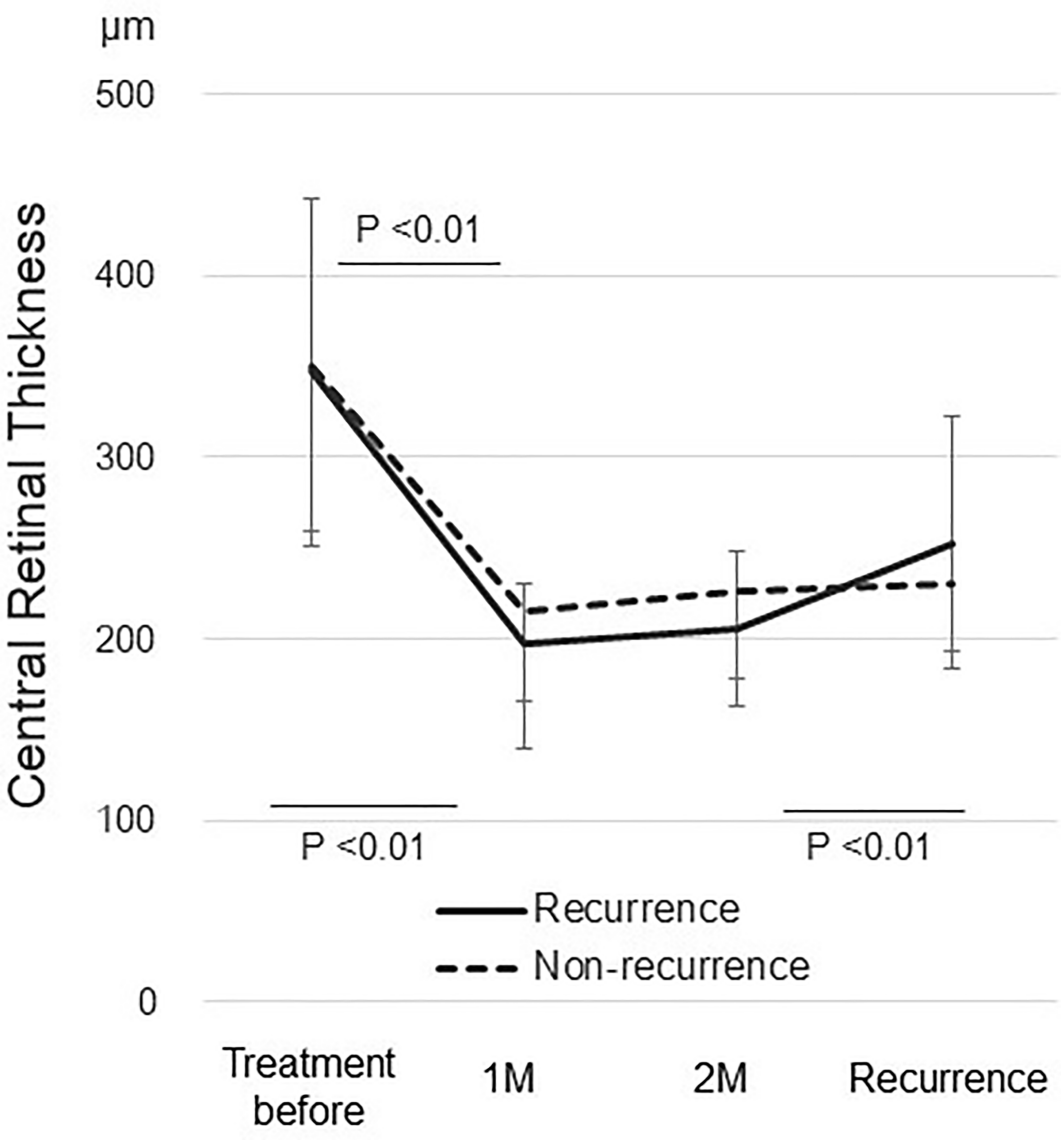
Changes of the CRT in all cases after aflibercept therapy. 1 M = 1 month after the loading phase; 2 M = 2 months after the loading phase.

In the 13 eyes with a recurrence, the mean sub-RPE area increased significantly (*P* = 0.036) from 2.03 ± 2.29 mm^2^ 1 month after the loading phase to 2.42 ± 2.08 mm^2^ 2 months after the loading phase. The mean sub-RPE volume increased marginally (*P* = 0.06) from 0.12 ± 0.17 mm^3^ 1 month after the loading phase to 0.15 ± 0.17 mm^3^ 2 months after the loading phase (Fig 2). In nine eyes without a recurrence, no significant changes were seen in the sub-RPE area or volume between 1 and 2 months after the loading phase (Fig 2). No significant changes in the CRT were seen between 1 and 2 months after the loading phase in the eyes with or without a recurrence (Fig 3).

**Fig 4:**
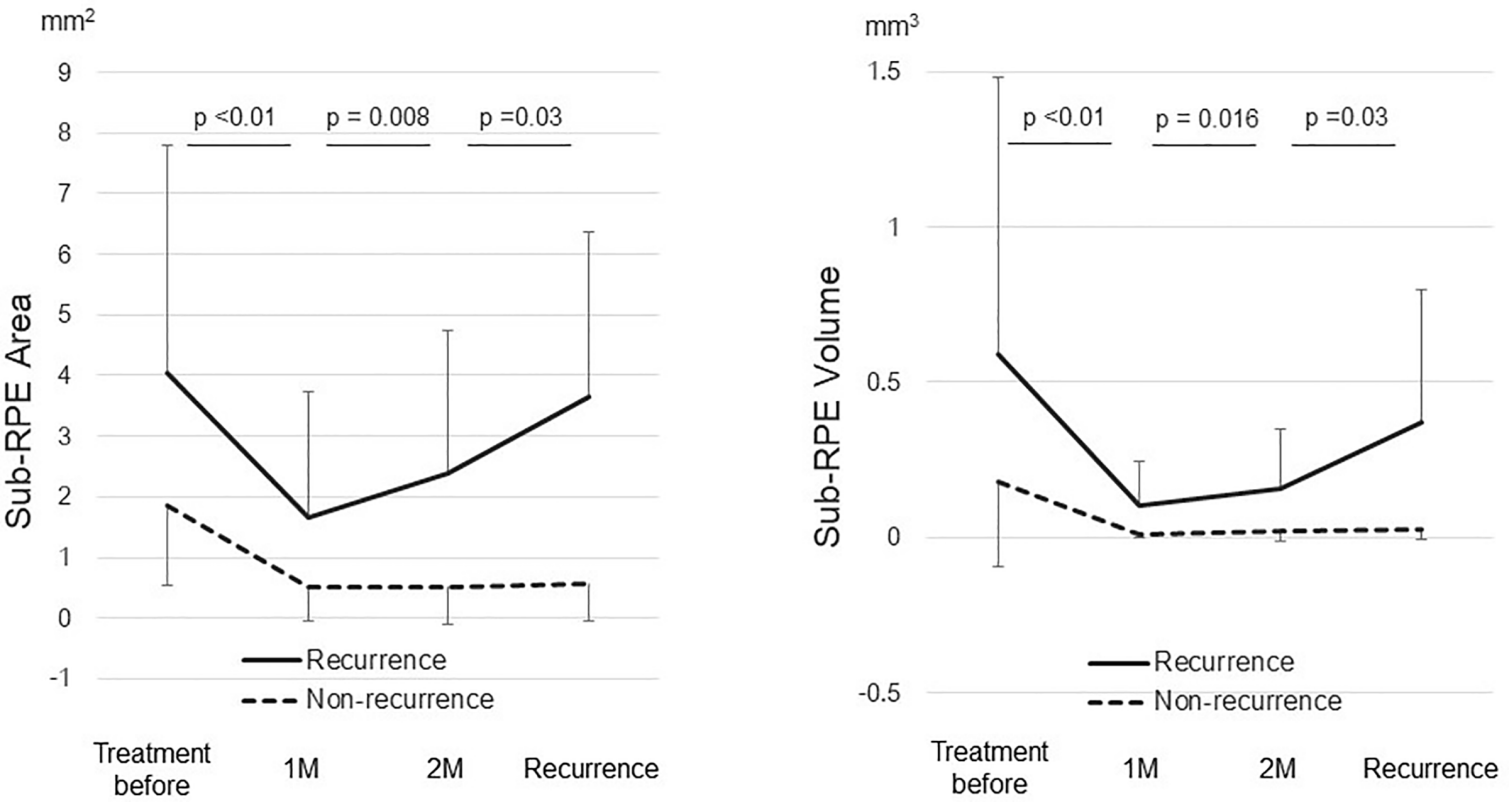
Changes of the sub-RPE area and volume in eyes with typical AMD after aflibercept therapy. 1 M = 1 month after the loading phase; 2 M = 2 months after the loading phase.

A certain inclination was found in eyes with typical AMD, but not in eyes with PCV or Rap in sub-RPE space between 1 and 2 months after the loading phase (S2 Table and S1 and S2 Figs), and the calculated statistical power was 0.85 for the recurrent cases with typical AMD So that, the changes in the sub-RPE area and volume were illustrated in eyes with typical AMD. In eyes with typical AMD, the mean sub-RPE area in eight eyes with a recurrence increased significantly (*P* = 0.008) from 1.66 ± 2.08 mm^2^ 1 month after the loading phase to 2.38 ± 2.35 mm^2^ 2 months after the loading phase. The mean sub-RPE volume increased significantly (*P* = 0.016) from 0.10 ± 0.14 mm^3^ 1 month after the loading phase to 0.16 ± 0.20 mm^3^ 2 months after the loading phase (Fig 4). In four eyes with typical AMD without a recurrence, no significant changes were found in the sub-RPE area or volume between 1 and 2 months after the loading phase (Fig 4). There were no significant changes in the CRT between 1 and 2 months after the loading phase either in eyes with or without a recurrence.

Further analysis of eyes with typical AMD showed that all sub-RPE areas in eight of eight eyes with a recurrence increased from 1 to 2 months after the loading phase, whereas those in three of the four eyes without a recurrence did not increase. The positive and negative predictive values were obtained to associate the increases in the sub-RPE areas from 1 to 2 months after the loading phase with recurrences. When the sub-RPE area increased from 1 to 2 months after the loading phase, the positive predictive value of a recurrence of AMD within 6 months was 0.89 (95% confidence interval [CI] 0.52–1.0), while the negative predictive value was 1.0 (95% CI, 0.29–1.0).

## Discussion and conclusion

The current study documented the detailed characteristics of the sub-RPE space and sensory retinas in eyes with neovascular AMD. An advanced quantitative analysis demonstrated an increased likelihood of sub-RPE area and volume between 1 and 2 months after the loading phase in eyes with recurrent AMD. Moreover, there could be an association between the increased sub-RPE area and a recurrence in eyes with typical AMD.

Previous studies have proposed that changes in the sub-RPE spaces potentially reflect the primary disease activity in both serous and vascularized PEDs.[4, 5] In the VIEW2 study, Schmidt-Erfurth et al.[4] reported that eyes with a primary serous PED and a persistent or recurrent PED were likely to have intraretinal cystic formation and progressive visual loss in the second year after switching from monthly injections to the PRN regimen. This result suggested that the presence of a serous PED might reflect the activity of the primary disease. In a case series of 14 eyes with vascularized PEDs, Penha et al.[5] reported that treatment was needed at the next visit whenever the volume of the vascularized PED increased compared with that during previous visits, while treatment was not needed at the next visit when there was no increase. In the current study, the sub-RPE space was likely to increase shortly after the loading phase in eyes with a recurrence within 6 months, and there was no significant change in the CRT in eyes with or without a recurrence. Although the sample size was too small to conclude, the current results could support the previously reported findings, and demonstrated that the changes in the sub-RPE space could be more sensitive markers than the sensory retinal changes for monitoring the disease activity. Moreover, the current study included only 2 eyes with vascularized PED, which suggests that this idea could also be applied to pathologies of other sub-types of neovascular AMD.

Regarding to subtypes, eyes with typical AMD had an increased likelihood of the sub-RPE space shortly after the loading phase in the recurrence group, while those changes were not seen in eyes with PCV or RAP; although the sample sizes by subtypes were too small to reach the conclusion. The pathological differences among the AMD subtypes might be involved in the different modes of development of the sub-RPE space. [1, 3, 10, 13]

Arguments regarding a suitable treatment regimen for neovascular AMD remain and aim for individualized medicine. The SUSTAIN study reported that 20.5% of the participants needed no additional injections after three consecutive injections during the first year, and 33.2% of the participants needed only one or two additional injections using the PRN regimen[14]. Although the PRN regimen often results in a visual acuity loss during long-term follow-up compared with monthly treatment,[14–17] these 53.7% of participants maintained improved vision[14]. The treat-and-extend (TandE) regimen should preserve vision as well as the monthly regimen by providing a planned injection before a recurrence[18]. However, considering the fact that the TandE regimen needs at least four additional injections during the first year [19], the PRN regimen might be more beneficial for over a half of AMD patients who needs less than 3 additional treatments following the advanced quantitative analysis of the sub-RPE space. Although the current study size was small and prospective large-scale studies are warranted to validate this observation, our findings might help predict recurrences with the goal of designing individualized treatment regimens based on the recurrent frequency in the early phase of treatment. This eventually would lead to more sophisticated individualized management of neovascular AMD.

There are limitations in the current study. First, monthly measurements were not available for many patients using the PRN regimen, so the sample size of the current study was not sufficiently large to obtain adequate statistical power. Therefore, future studies to validate these findings are needed to exclude the possibility of a chance finding. Second, eyes with extensive PEDs or fibrosis could not be segmented and/or measured appropriately by the software and were therefore excluded. A new segmentation technique is needed to examine such cases in the future.

In conclusion, we demonstrated that eyes with recurrent AMD had an increased likelihood of the sub-RPE space shortly after the loading phase. These changes could indicate disease activity and might be predictive of recurrences, which could be useful to design individualized regimens in the early phase of the treatment.

## Supporting information

S1 Table Patient characteristics. (DOCX)

S2 Table Retinal and sub-retinal pigment epithelial space measurements in all cases during the 6-month follow-up period. (DOC)

S1 Fig Changes of the sub-RPE area in respective cases with typical AMD. (TIF)

S2 Fig Changes of the sub-RPE area in respective cases with PCV or Rap. (TIF)
