# Supplementary material for: Advanced quantitative analysis of the sub-retinal pigment epithelial space in recurrent neovascular age-related macular degeneration
Source: PLoS One. 2017 Nov 2;12(11):e0186955. doi: 10.1371/journal.pone.0186955 (PMC5667874; doi:10.1371/journal.pone.0186955)
Supplement: S1 Table — (DOCX) [file pone.0186955.s001.docx]

S1 Table. Patient Characteristics

|  |  | Patients | No Recurrence  (A) | Recurrence  (B) | *P* for Difference  (A vs. B) |
| --- | --- | --- | --- | --- | --- |
| No. eyes |  | 22 | 9 | 13 |  |
| Age, years |  | 74.2 ± 8.9 | 71.1 ± 8.7 | 77.3 ± 7.9 | 0.20 |
| Sex, % men |  | 63.6 | 55.6 | 69.2 | 0.66 |
| AMD subtype,  No. (%) | Typical AMD | 12 (54.5) | 4 (44.4) | 8 (61.5) | 0.25 |
|  | PCV | 7 (31.8) | 4 (44.4) | 3 (23.1) |  |
|  | RAP | 3 (13.6) | 1 (11.1) | 2 (15.4) |  |
| Quality assessment | SRF | 20 (90.9) | 9 (100) | 11 (84.6) | 0.49 |
|  | IRF | 9 (40.9) | 4 (44.4) | 5 (38.5) | 1.0 |
|  | PED | sPED 5/ hPED 1/ fPED 2  (36.4) | sPED 3/  hPED 1  (44.4) | sPED 2/  fPED 2  (30.8) | 0.66 |
| Injection numbers to d  dry macula |  | 3.1± 0.6 | 3.0 | 3.2 ± 0.8 | 0.34 |
| CRT,  μm |  | 348.3 ± 91.7 | 349.6 ± 91.0 | 347.3 ± 95.9 | 0.10 |
| Sub-RPE area,  mm^2^ |  | 3.73 ± 2.99 | 2.98 ± 2.44 | 4.25 ± 3.31 | 0.67 |
| Sub-RPE volume,  mm^3^ |  | 0.50 ± 0.65 | 0.33 ± 0.36 | 0.62 ± 0.78 | 0.46 |

sPED: serous PED, fPED: fibrovascular PED, hPED: hemorrhagic PED

The values are expressed as the mean and standard deviation for continuous variables or as percentages for categories.

AMD, age-related macular degeneration; CRT, central retinal thickness; PCV, polypoidal choroidal vasculopathy; RAP, retinal angiomatous proliferation; RPE, retinal pigment epithelial
