# Supplementary material for: Advanced quantitative analysis of the sub-retinal pigment epithelial space in recurrent neovascular age-related macular degeneration
Source: PLoS One. 2017 Nov 2;12(11):e0186955. doi: 10.1371/journal.pone.0186955 (PMC5667874; doi:10.1371/journal.pone.0186955)
Supplement: S2 Table — (DOC) [file pone.0186955.s002.doc]

**S2 Table. Retinal and Sub-Retinal Pigment Epithelial Space Measurements during the 6-Month Follow-Up Period**

|  |  |  |  |  |  | Central Retinal Thickness (μｍ) | | | | Sub-RPE Area (mm^2^) | | | | Sub-RPE Volume (mm^3^) | | | | |
| --- | --- | --- | --- | --- | --- | --- | --- | --- | --- | --- | --- | --- | --- | --- | --- | --- | --- | --- |
| Recurrent cases | Age | Sex | AMD sub-type | recurrence | Time to recurrence  (Months) | Baseline | 1M | 2M | Recurrence | Baseline | 1M | 2M | Recurrence | Baseline | | 1M | 2M | Recurrence |
| 1 | 80 | F | tAMD | 1 | 2 | 495 | 221 | 253 | 253 | 0.4 | 0.2 | 0.3 | 0.3 | 0.02 | 0.01 | | 0.01 | 0.01 |
| 2 | 64 | M | tAMD | 1 | 4 | 285 | 228 | 227 | 245 | 2.2 | 1.1 | 1.7 | 3.9 | 0.11 | 0.04 | | 0.06 | 0.22 |
| 3 | 82 | M | tAMD | 1 | 5 | 270 | 175 | 175 | 251 | 1.5 | 0.3 | 0.9 | 1.0 | 0.08 | 0.01 | | 0.03 | 0.05 |
| 4 | 71 | M | tAMD | 1 | 3 | 273 | 194 | 210 | 408 | 1.2 | 0.01 | 0.6 | 0.6 | 0.04 | 0 | | 0.02 | 0.02 |
| 5 | 69 | F | tAMD | 1 | 3 | 337 | 182 | 186 | 202 | 2 | 0 | 0.3 | 1.2 | 0.07 | 0 | | 0.01 | 0.04 |
| 6 | 87 | M | tAMD | 1 | 3 | 359 | 236 | 234 | 282 | 7.9 | 5.8 | 6.2 | 6.9 | 1.57 | 0.4 | | 0.44 | 1.05 |
| 7 | 80 | M | tAMD | 1 | 3 | 270 | 160 | 163 | 176 | 10.4 | 2.8 | 5.1 | 6.4 | 2.37 | 0.2 | | 0.47 | 0.9 |
| 8 | 75 | F | tAMD | 1 | 3 | 241 | 187 | 185 | 227 | 6.8 | 3.1 | 3.9 | 5.6 | 0.47 | 0.16 | | 0.21 | 0.32 |
| 9 | 78 | M | PCV | 1 | 5 | 497 | 134 | 132 | 162 | 5.6 | 2.6 | 1.6 | 1.1 | 1.75 | 0.13 | | 0.06 | 0.03 |
| 10 | 75 | M | PCV | 1 | 2 | 304 | 213 | 290 | 290 | 1.7 | 0.2 | 1.4 | 1.4 | 0.15 | 0.01 | | 0.07 | 0.07 |
| 11 | 68 | M | PCV | 1 | 3 | 267 | 197 | 181 | 214 | 7.1 | 2.7 | 2.7 | 5.2 | 0.7 | 0.11 | | 0.11 | 0.32 |
| 12 | 87 | F | RAP | 1 | 3 | 470 | 251 | 248 | 347 | 7.1 | 7.2 | 5.4 | 5.9 | 0.67 | 0.53 | | 0.41 | 0.39 |
| 13 | 89 | M | RAP | 1 | 4 | 447 | 192 | 193 | 280 | 1.4 | 2.1 | 2.3 | 3.3 | 0.05 | 0.07 | | 0.08 | 0.15 |
| Mean | 77.3 |  |  |  | 3.3 | 347.3 | 197.7 | 205.9 | 256.7 | 4.3 | 2.0 | 2.4 | 3.3 | 0.62 | 0.12 | | 0.15 | 0.27 |
| SD | 7.9 |  |  |  | 0.95 | 95.9 | 32.2 | 43.0 | 67.5 | 3.3 | 2.3 | 2.1 | 2.5 | 0.78 | 0.17 | | 0.17 | 0.34 |
|  |  |  |  |  | | Central Retinal Thickness (μｍ) | | | | Sub-RPE Area (mm^2^) | | | | Sub-RPE Volume (mm^3^) | | | | |
| Non-recurrent cases | Age | Sex | AMD sub-type | recurrence | Time to recurrence  (Months) | Baseline | 1M | 2M | 6M | Baseline | 1M | 2M | 6M | Baseline | 1M | | 2M | 6M |
| 14 | 67 | M | tAMD | 0 | NA | 464 | 234 | 234 | 244 | 2.9 | 1.3 | 1.3 | 1.6 | 0.58 | 0.03 | | 0.07 | 0.11 |
| 15 | 74 | M | tAMD | 0 | NA | 293 | 198 | 194 | 206 | 2.7 | 0.4 | 0.6 | 1.2 | 0 | 0.01 | | 0.02 | 0.04 |
| 16 | 75 | F | tAMD | 0 | NA | 498 | 239 | 246 | 251 | 1.8 | 0 | 0 | 0.01 | 0.14 | 0 | | 0 | 0 |
| 17 | 52 | F | tAMD | 0 | NA | 444 | 234 | 232 | 235 | 0 | 0.3 | 0.1 | 0.3 | 0 | 0.01 | | 0 | 0.02 |
| 18 | 80 | M | PCV | 0 | NA | 282 | 258 | 258 | 260 | 2.5 | 1.9 | 1 | 0.3 | 0.16 | 0.21 | | 0.11 | 0.01 |
| 19 | 64 | F | PCV | 0 | NA | 289 | 288 | 289 | 293 | 3.8 | 0.9 | 0.8 | 0.4 | 0.54 | 0.05 | | 0.04 | 0.01 |
| 20 | 75 | M | PCV | 0 | NA | 317 | 219 | 220 | 217 | 3.6 | 2.3 | 2.3 | 1.7 | 0.45 | 0.12 | | 0.13 | 0.07 |
| 21 | 77 | M | PCV | 0 | NA | 280 | 246 | 248 | 252 | 0.9 | 0.6 | 0.4 | 1 | 0.05 | 0.02 | | 0.01 | 0.04 |
| 22 | 76 | F | RAP | 0 | NA | 280 | 119 | 157 | 227 | 8.6 | 3.9 | 4 | 4.6 | 1.08 | 0.88 | | 0.76 | 0.79 |
| Mean | 71.1 |  |  |  |  | 349.7 | 226.1 | 230.9 | 242.8 | 3.0 | 1.3 | 1.2 | 1.2 | 0.33 | 0.15 | | 0.13 | 0.12 |
| SD | 8.7 |  |  |  |  | 91.0 | 47.2 | 38.1 | 25.7 | 2.4 | 1.2 | 1.3 | 1.4 | 0.36 | 0.28 | | 0.24 | 0.25 |

1 M, 1 month after the loading phase; 2 M, 2 months after the loading phase; 6 M, 6 months after loading phase; F, female; M, male; sub-RPE, subretinal age-related macular degeneration; CRT, central retinal thickness; tAMD, typical age-related macular degeneration; PCV, polypoidal choroidal vasculopathy; RAP, retinal angiomatous proliferation; SD, standard deviation; NA, not applicable.
