# Supplementary figures and images for: Advanced quantitative analysis of the sub-retinal pigment epithelial space in recurrent neovascular age-related macular degeneration
Source: PLoS One. 2017 Nov 2;12(11):e0186955. doi: 10.1371/journal.pone.0186955 (PMC5667874; doi:10.1371/journal.pone.0186955)

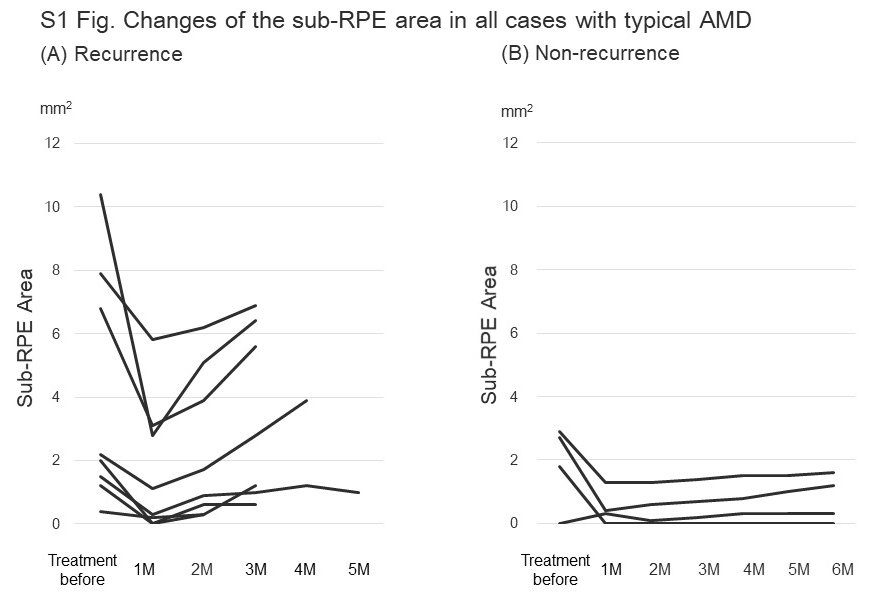

Supplement: S1 Fig — (TIF) [file pone.0186955.s003.tif]

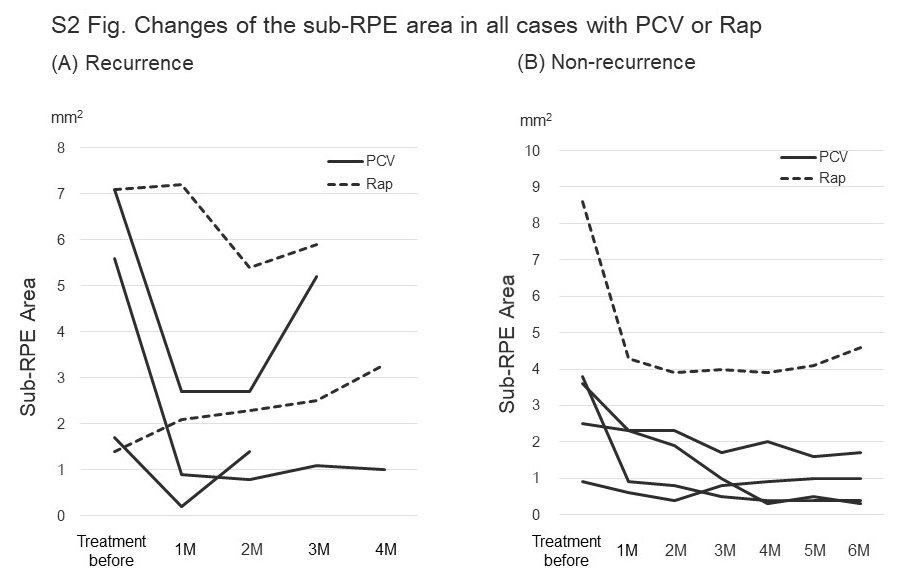

Supplement: S2 Fig — (TIF) [file pone.0186955.s004.tif]
